# Sero-Positive Isolated Ocular Myasthenia Gravis

**DOI:** 10.7759/cureus.66885

**Published:** 2024-08-14

**Authors:** Taimoor A Khan, Muhammad Zeshan Sadiq, Ali A Khan, Muhammad A Zahid, Sheharyar Zameer

**Affiliations:** 1 Ophthalmology, National University of Medical Sciences (NUMS) Rawalpindi, Rawalpindi, PAK; 2 Ophthalmology, Armed Forces Institute of Ophthalmology Rawalpindi, Rawalpindi, PAK; 3 General Medicine, Military Hospital Rawalpindi, Rawalpindi, PAK; 4 Internal Medicine, Ayub Medical College, Abbottabad, PAK; 5 Ophthalmology, Monash Health, Clayton, AUS; 6 General Surgery, Fauji Foundation Hospital, Rawalpindi, PAK

**Keywords:** seropositivity, myasthenia gravis, neuromuscular diseases, autoimmune diseases, polyneuropathy

## Abstract

Ocular myasthenia gravis (OMG) is a neuromuscular disease characterized by the production of autoantibodies against post-synaptic proteins at the neuromuscular junction (NMJ). An 18-year-old male who had symptoms of drooping eyelids and double vision was diagnosed with ocular myasthenia gravis on investigations and examinations. Treatment was initiated with a tablet of pyridostigmine 60 mg twice daily per oral for two weeks, followed by three times daily for four weeks. The patient demonstrated significant improvement in ptosis and diplopia. There are still a considerable number of challenges in the diagnosis and treatment of ocular myasthenia gravis, with the typical treatment involving acetylcholinesterase inhibitors and immunosuppressants.

## Introduction

Myasthenia gravis (MG) is an autoimmune disorder that is distinguished by fluctuating muscle weakness, predominantly impacting the neuromuscular junction (NMJ) of skeletal muscles [1]. Ocular myasthenia gravis (OMG) is a specific form of MG characterized by the presence of localized weakness in the extraocular muscles (EOMs), levator palpebrae superioris (LPS) complex, and orbicularis oculi, while not affecting other skeletal muscles [2]. This case report details a patient who exclusively exhibits ocular myasthenia gravis, encompassing an analysis of their clinical manifestation, the difficulties encountered during diagnosis, and the strategies employed for management.

Several factors can be attributed to the distinctive vulnerability of extraocular muscles to myasthenia gravis. Extraocular muscles are composed of twitch fibers that exhibit a greater rate of tension development and a higher frequency of synaptic firing than limb muscles. Consequently, extraocular muscles are more susceptible to fatigue. Furthermore, it has been observed that tonic muscle fibers, which play a vital role in sustaining gaze in various directions, exhibit a lower density of acetylcholine receptors. This characteristic renders them more vulnerable to the loss or impairment of these receptors [3]. Furthermore, extrinsic ocular muscles display differential expression of multiple genes, including those related to the immune response, which sets them apart from conventional skeletal muscles [4].

Patients who have received a diagnosis of oculomotor nerve palsy frequently demonstrate fluctuating degrees of weakness in the extraocular muscles, a condition that generally improves after periods of rest. Respiratory muscle contractions exacerbate the weakness [2]. Ptosis, which is characterized by the drooping of the eyelids, and diplopia, leading to the perception of double vision, are the predominant initial symptoms observed in more than 50% of patients diagnosed with myasthenia gravis [5]. A considerable proportion, ranging from 50% to 80%, of individuals who experience ocular symptoms eventually develop generalized myasthenia gravis (GMG). It is worth mentioning that the participation of various extraocular muscles gives rise to a range of movement patterns, which complicates the identification of precise nerve lesions responsible for the impairments [4].

The occurrence of ptosis in oculomotor nerve palsy is primarily attributed to the involvement of the levator palpebrae superioris complex. Unilateral or bilateral occurrence is possible, with the presence of asymmetry often noted in cases of bilateral manifestation. The "lid fatigability test" is a method used to observe the phenomenon of ptosis, which refers to the drooping of the upper eyelid. This test involves sustained upward gaze for an extended period, during which an observable increase in ptosis can be detected. One additional clinical manifestation, known as Cogan's lid twitch, is characterized by a rapid upward movement followed by a downward drift of the upper eyelid upon returning to the primary position after looking downward. Nevertheless, it should be noted that Cogan's lid twitch is not exclusive to ocular myasthenia gravis. Hering's law, a principle that guarantees equitable innervation to paired muscles, elucidates the exacerbation of ptosis in the eye opposite to the one being manually elevated, as observed in the act of lifting the drooping eyelid [6].

Diplopia, a common symptom in ocular myasthenia gravis, is observed even in cases of mild weakness of the extraocular muscles, as these muscles do not demonstrate the same level of adaptability to variable weakness as limb muscles [3,7]. Ocular myasthenia gravis has the ability to imitate diverse forms of comitant or incomitant strabismus patterns, encompassing nerve palsies, gaze palsies, and even complete ophthalmoplegia. Therefore, it is important to consider the possibility of oculomotor nerve palsy when evaluating patients with variable incomitant strabismus, with or without ptosis [3].

## Case presentation

**Figure 1:**
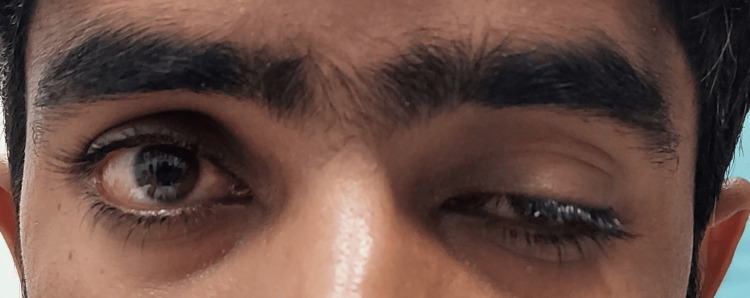
Severe left ptosis in primary gaze.

An 18-year-old male presented with a three-day history of new-onset left-sided ptosis and diplopia in the superior gaze. He had no known comorbidities and denied any history of snake bites or other significant exposures. A recent symptomatic but uncomplicated PCR-positive SARS-CoV-2 infection was the only significant medical history. On general physical examination, he appeared well-oriented with stable vital signs, and a systemic examination revealed no remarkable findings. Peripheral examination revealed no fang marks or signs of envenomation. Adnexal examination revealed severe left-sided ptosis and right scleral show due to frontalis over-action. Examination revealed decreased levator function, reduced marginal reflex distance (MRD1), and asymmetrical palpebral fissure height (Figure 1). The left eye demonstrated marked fatigability in sustained superior gaze.

**Figure 2:**
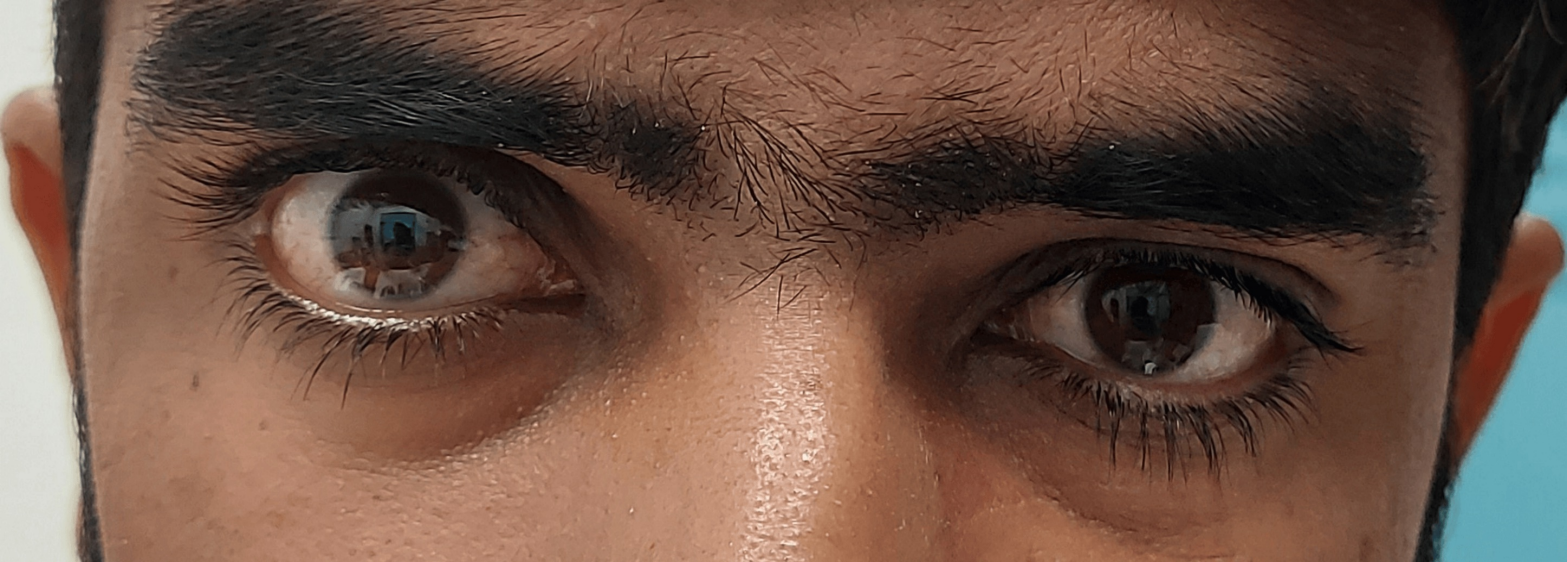
Improvement in ptosis after the two minute ice application test.

There was a good bell phenomenon in both eyes. The presence of a positive response with a dramatic improvement in ptosis to the two-minute ice application test further supported the clinical suspicion of myasthenia gravis (Figure 2). Binocular diplopia was observed in superior gaze, and pursuit movements showed limitations in levo-elevation. The Cogan Twitch sign was positive on vertical saccades. His provocative testing for systemic signs of myasthenia gravis was negative.

**Figure 3:**
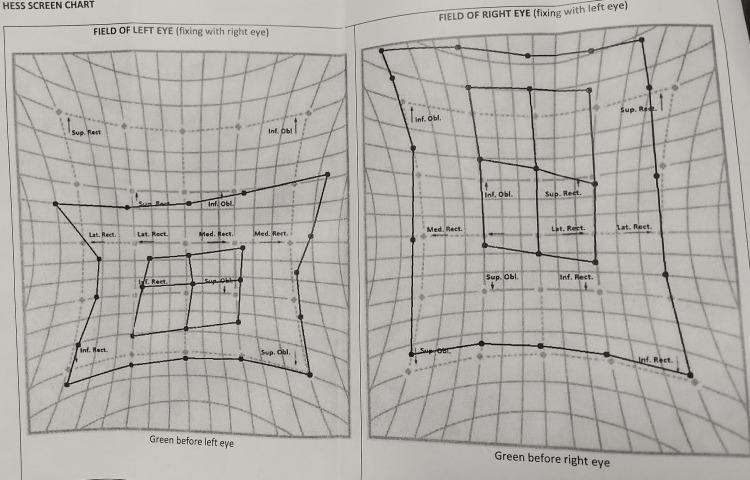
Baseline HESS screen chart.

**Figure 4:**
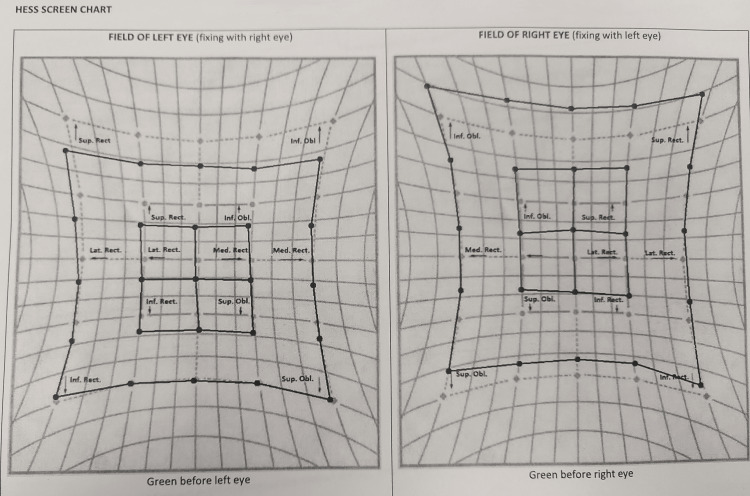
Follow-up HESS screen chart.

Baseline investigations, including routine laboratory tests, were regular. However, anti-Ach receptor antibodies were strongly positive (repeated on two occasions for confirmation), confirming the diagnosis of seropositive myasthenia gravis. Imaging studies were unremarkable, including an high-resolution computed tomography (HRCT) scan of the chest to rule out thymoma and a contrast-enhanced MRI of the brain and orbit. Thyroid function tests were within normal limits. Electromyography nerve conduction studies with repetitive nerve stimulation showed no decrement, which is atypical for myasthenia gravis. The patient was diagnosed with seropositive isolated ocular myasthenia gravis based on the clinical presentation, positive anti-Ach receptor antibodies, fatigability in sustained superior gaze, and positive response to the ice test. Treatment was initiated with a tablet of pyridostigmine 60 mg twice daily per oral for two weeks, followed by three times daily for four weeks. The patient demonstrated significant improvement in ptosis and diplopia. To further optimize his management, the patient was referred to a neurologist who prescribed a short course of systemic corticosteroids (tablet prednisolone enteric coated 5 mg, 16 tablets per oral, once daily, making a total dose of 80 mg once daily for five days) to provide immediate symptomatic relief. Maintenance therapy with the tablet mycophenolate mofetil (MMF) 500 mg twice daily per oral was initiated as a steroid-sparing agent. The patient showed remarkable improvement in ptosis and diplopia. Baseline and follow-up HESS screen charts were performed to track the improvement (Figures 3-4).

## Discussion

According to a study conducted in 2021, the annual incidence of myasthenia gravis varied between 4.1 and 30 cases per million individuals [8]. In women, there is a bimodal distribution that exhibits two distinct peaks occurring at approximately 30 and 50 years of age. In the male population, the prevalence of this phenomenon exhibits a consistent upward trend as individuals grow older, reaching its peak between the ages of 60 and 89 [9]. The prevalence rate of ocular myasthenia gravis varies from 150 to 200 cases per million individuals [10]. Over the past five decades, there has been a consistent upward trend in these rates, which can be attributed to advancements in the identification, clinical diagnosis, and treatment of ocular myasthenia gravis and an overall increase in life expectancy [10].

One of the most frequently observed symptoms upon presentation is diplopia, accompanied by orbicularis involvement. The individual exhibits symptoms of muscle weakness in the orbicularis muscle and ptosis. The symptoms mentioned above can potentially lead to the development of generalized myasthenia gravis in approximately 20-60% of cases involving the bulbar muscles. These symptoms include difficulties with chewing, frequent choking, dysarthria, dysphagia, hoarseness, weakness in facial muscles resulting in a lack of expression, and involvement of neck muscles, leading to a dropped-head syndrome. Additionally, axial and limb muscles may be affected, with a tendency for proximal muscles to be more affected than distal muscles. Within two years, approximately 50% of patients experience involvement of the upper limbs to a greater extent than the lower limbs [11].

The primary method for diagnosing myasthenia gravis is through clinical evaluation. Clinical testing encompasses various diagnostic procedures, including the ice test, which has been reported to exhibit a sensitivity of approximately 90% and a specificity of 100% [12]. Additionally, the sleep and rest test has also been utilized for diagnostic purposes. The utilization of serological assays in the identification of acetylcholine receptors, muscle-specific tyrosine kinase, and low-density lipoprotein 4 has proven to be advantageous. However, the precise diagnostic significance of these assays remains uncertain. Consequently, clinical tests such as the ice and electrophysiological tests have played a crucial role in establishing a reliable diagnosis. Electrophysiological tests, such as repetitive nerve stimulation tests, demonstrate a decremental response in GMG. However, it is worth noting that this abnormality is observed in approximately 30-50% of cases of ocular myasthenia gravis [11].

The absence of randomized controlled trials and the infrequent manifestation of the disease have contributed to the absence of a universally agreed-upon treatment algorithm for myasthenia gravis. The primary therapeutic approach is implemented in collaboration with the field of neurology and centres on the utilization of acetylcholinesterase inhibitors and steroids, intravenous immunoglobulin, secondary immunosuppressants, and plasma exchange. The standard approach to treatment typically involves the administration of cholinesterase inhibitors, which have demonstrated efficacy in the management of ptosis. Additionally, the use of steroids has been shown to be highly effective in addressing the simultaneous occurrence of diplopia and ptosis [13]. Surgical interventions, such as ptosis repair, strabismus repair, and thymectomy, are considered after achieving optimal medical management. Clinicians make decisions regarding the implementation of additional medical treatment modalities based on the progression and severity of the disease. Subsequent evaluation after the commencement of treatment involves the utilization of dependable biomarkers to evaluate the efficacy of treatment and the severity of the disease.

There is evidence to suggest that a significant portion of individuals diagnosed with ocular myasthenia gravis possess antibodies targeting acetylcholine receptors. Multiple hypotheses have been proposed to explain the preferential involvement of extraocular muscles in ocular myasthenia gravis. The extraocular muscles exhibit a propensity for fatigue due to their reliance on tonic contractions to maintain prolonged fixation in a specific direction. Additionally, these muscles possess fibres that demonstrate a heightened frequency of synaptic firing and a more rapid development of tension. Moreover, the extraocular muscles exhibit a comparatively reduced density of acetylcholine receptors, rendering them more susceptible to experiencing symptoms of fatigue. It is also hypothesized that contrasting epitope expression in extraocular muscles plays a role in their preferential involvement [8].

The prognosis of myasthenia gravis is contingent upon various factors, such as gender, age at onset, outcomes of electrophysiological examinations, the presence and quantity of antibodies targeting acetylcholine receptors, therapeutic interventions, and abnormalities in the thymus. The prognosis for myasthenia gravis, both ocular and systemic, is considered favourable when symptoms are effectively treated and managed and there is no substantial progression of the disease to affect larger muscle groups, particularly the bulbar muscles responsible for respiratory function and swallowing [14].

## Conclusions

In conclusion, this case report highlights the unique clinical presentation of unilateral ptosis, diagnostic challenges, and management strategies associated with ocular myasthenia gravis. The successful management of the patient involved the initiation of oral pyridostigmine, followed by systemic corticosteroids and mycophenolate mofetil. The multidisciplinary approach, incorporating neurology and ophthalmology expertise, contributed to the notable improvement in ptosis and diplopia symptoms. This report emphasizes that with early recognition and appropriate management, patients with ocular myasthenia gravis can achieve favorable outcomes and delay progression to generalized myasthenia gravis.
